# Integrated microbiome-metabolome analysis reveals multiorgan toxicity of 1-nitropyrene and the limited efficacy of ferroptosis inhibitor Fer-1 in rats

**DOI:** 10.3389/ftox.2026.1771766

**Published:** 2026-03-13

**Authors:** Ning Yu, Shuguang Pang, Youmei Li, Hongcui Diao

**Affiliations:** 1 Department of Endocrinology, Jinan Central Hospital, Shandong University, Jinan, China; 2 Department of Endocrinology, Zibo Central Hospital, Zibo, China; 3 Department of Endocrinology, Jinan Central Hospital Affiliated to Shandong First Medical University, Jinan, China; 4 Department of Endocrinology, Changqing District People’s Hospital, Jinan, China

**Keywords:** 1-nitropyrene, blood glucose, ferroptosis, metabolic, non-alcoholic fatty liver disease

## Abstract

**Introduction:**

1-Nitropyrene (1-NP), a prevalent nitro-polycyclic aromatic hydrocarbon, is increasingly recognized as a potential metabolic disruptor, yet its systemic biological effects remain insufficiently characterized.

**Methods:**

This study investigated the metabolic, immunological, hepatic, and microbiome alterations induced by chronic 1-NP exposure in rats and assessed whether ferroptosis inhibition via Fer-1 could mitigate these effects.

**Results:**

Although body weight was not significantly altered overall, high-dose exposure impaired growth from week 4. Exposed groups exhibited progressively elevated fasting blood glucose and impaired glucose tolerance, indicating significant disruption of glucose homeostasis. Serum biochemistry revealed dose-dependent reductions in HDL and total cholesterol, while histopathology confirmed hepatocyte ballooning, inflammation, and steatosis consistent with NAFLD-like progression. Hematological changes, including shifts in neutrophil and lymphocyte populations, suggested chronic inflammatory activation. Untargeted metabolomics identified extensive alterations in pathways related to glycolysis, tryptophan metabolism, glycerophospholipid metabolism, and ABC transporters. Gut microbiota analysis demonstrated reduced richness and significant compositional shifts, with functional predictions linking dysbiosis to xenobiotic degradation, lipid metabolism, and phosphotransferase systems. Integrated microbiome–metabolome analysis revealed coordinated disruptions in host–microbial metabolic networks. Fer-1 intervention modified specific metabolic and microbial signatures but did not substantially alleviate major toxic outcomes.

**Conclusion:**

Overall, chronic 1-NP exposure causes widespread metabolic injury driven by combined effects on host metabolism, immune regulation, hepatic function, and gut microbial ecology. These findings highlight 1-NP as a potent environmental metabolic disruptor and underscore the need for further mechanistic studies to inform mitigation strategies.

## 1 Introduction

Environmental pollutants, particularly those derived from combustion processes such as diesel exhaust, pose significant risks to public health. Among these, 1-nitropyrene (1-NP) is a nitroaromatic compound classified as a probable human carcinogen by the International Agency for Research on Cancer (IARC) in 1998 (National_Toxicology_Program, 1998). It is abundantly present in urban air pollution and has been linked to respiratory (Koike et al., 2014; Sarver et al., 2019), cardiovascular (Zhao et al., 2019), and metabolic disorders (Shaoyong et al., 2023) in epidemiological studies. Chronic exposure to 1-NP occurs through inhalation, ingestion, and dermal contact, leading to bioaccumulation in tissues and systemic effects (Medinsky et al., 1989). The metabolism of 1-NP is primarily catalyzed by cytochrome P450 enzymes (Hatanaka et al., 2001), with CYP 2A13 and 2E1 being the key isoforms due to their superior binding affinity (Wang M. et al., 2023). It proceeds mainly via epoxidation and hydroxylation (Hatanaka et al., 2001). The epoxidation pathway is more favorable, featuring a lower energy barrier and generating 4,5-epoxide-1-NP and 9,10-epoxide-1-NP as major products through electrophilic addition (Fifer et al., 1986). Alternatively, hydroxylation yields 6-hydroxy-1-NP and 8-hydroxy-1-NP (Miller-Schulze et al., 2016). These metabolic products, especially the electrophilic epoxides that may bind to DNA, along with 1-NP itself, are associated with potential toxic effects on the lung and gastrointestinal system (Ritter et al., 2002). Recent research has shifted focus from its genotoxic properties to its role in inducing oxidative stress (Yun et al., 2019; Zhao et al., 2019; Choi S. H. et al., 2023) and metabolic reprogramming (Wang B. et al., 2023), which may underlie conditions like insulin resistance, dyslipidemia, and non-alcoholic fatty liver disease (NAFLD).

Ferroptosis, first described in 2012 (Dixon et al., 2012), is a regulated form of cell death distinct from apoptosis, necrosis, or autophagy. It is driven by iron accumulation, lipid peroxidation, and the failure of antioxidant defenses, particularly the glutathione (GSH)-dependent enzyme GPX4 (Cui et al., 2022; Zhang et al., 2024). Emerging evidence indicates that ferroptosis is implicated in metabolic diseases, including type 2 diabetes mellitus (T2DM) (Sha et al., 2021; Miao et al., 2023), obesity (Wang et al., 2025), and NAFLD (Cheng et al., 2023), where it exacerbates tissue damage through oxidative lipid damage and mitochondrial dysfunction. Pollutants like fine particulate matter (PM_2.5_) (Yan et al., 2022; Li et al., 2024) and heavy metals (Gong et al., 2025) have been shown to induce ferroptosis in animal models, leading to organ-specific injuries. However, the specific involvement of ferroptosis in 1-NP-induced metabolic perturbations remains underexplored.

The metabolic system, encompassing glucose homeostasis, lipid metabolism, and energy balance, is highly susceptible to environmental pollutants (Formichi et al., 2025). In rodents, chronic pollutant exposure disrupts hepatic glucose metabolism (Ding et al., 2025), impairs insulin signaling (Gong et al., 2024), and promotes steatosis (Qin et al., 2022). NAFLD, characterized by hepatic fat accumulation without excessive alcohol consumption, is a hallmark of metabolic syndrome (Brunt et al., 2015) and is increasingly linked to organic pollutants (Rantakokko et al., 2015; Du et al., 2025). Furthermore, the gut microbiome plays a crucial role in modulating host metabolism (Jyoti and Dey, 2025) through the production of short-chain fatty acids (SCFAs) (Han et al., 2025; Mukhopadhya and Louis, 2025), bile acid metabolism (Zhao et al., 2023), and immune regulation (Yang and Cong, 2021). Dysbiosis induced by toxins can alter metabolomic profiles, exacerbating systemic inflammation and metabolic disorders (Mostafavi Abdolmaleky and Zhou, 2024).

This study aims to elucidate the mechanistic role of ferroptosis in 1-NP’s effects on SD rat metabolism. Through a chronic exposure model, we assessed glucose parameters, liver pathology, intestinal metabolomics, and gut microbiota composition. We hypothesize that 1-NP triggers ferroptosis in hepatic and intestinal cells, leading to metabolic dysregulation, with gut microbiome shifts amplifying these effects. Understanding these pathways could inform interventions for pollution-related metabolic diseases.

## 2 Materials and methods

### 2.1 Animal model and exposure protocol

Male Sprague-Dawley rats (n = 40, aged 6–8 weeks, weight 200–250 g) were obtained from a certified breeder and housed under standard conditions (22 °C ± 2 °C, 12-h light/dark cycle, *ad libitum* access to chow and water). After a 1-week acclimation, 8 rats were randomly divided into control (vehicle: corn oil) and 1-NP exposure groups (low dose: 0.5 mg/kg; high dose: 2 mg/kg body weight) (Bostrom et al., 2002; Yu X. et al., 2022; Yu et al., 2024). To investigate the role of ferroptosis in the toxicity of 1-NP, we additionally designed two intervention groups using the ferroptosis inhibitor Fer-1, namely: the Con + Fer-1 group, in which Fer-1 was administered subcutaneously under the same conditions as the control group, and the High + Fer-1 group, in which Fer-1 was administered subcutaneously under the same conditions as the high-dose group. The intervention dosage of Fer-1 (Ferrostatin-1, 3-amino-4-(cyclohexylamino)-benzoic acid ethyl ester, Cayman Chemical Co., United States) was 5 mg/kg body weight, administered according to the manufacturer’s instructions. 1-NP (purity >98%, Sigma-Aldrich) was administered via oral gavage 5 days per week for 3 months, mimicking chronic environmental exposure. Body weights were monitored weekly, and ethical approval was obtained from the institutional animal care committee of Tianjin Jinke Bona Biotechnology (GENINK-20230031).

### 2.2 Glucose metabolism assessments

Fasting blood glucose (FBG) was measured using a glucometer (Accu-Chek, Roche) after 12-h fasting at baseline, month 1, 2, and 3. Oral glucose tolerance tests (OGTT) were performed at the end of exposure: rats received 2 g/kg glucose orally, and blood glucose was sampled at 0, 30, 60, 90, 120, and 180 min. Area under the curve (AUC) was calculated using the trapezoidal rule (Sakaguchi et al., 2016).

$$\text{AUC}=\Sigma \;\left[\;\left({\text{BG}}_{n}+{\text{BG}}_{n+1}\right)\;/\;2\;\right]\times \left(t_{n+1}-t_{n}\right)$$

The index n (0, 1, 2, 3, 4, 5, 6) corresponds to measurement times of 0, 30, 60, 90, 120, and 180 min, where t_n_ is the specific time of the nth measurement.

### 2.3 Liver histopathology and NAFLD scoring

Post-exposure, livers were excised, weighed, and fixed in 10% formalin. Paraffin-embedded sections (5 μm) were stained with hematoxylin-eosin (H&E) and Oil Red O for lipid accumulation. NAFLD activity score (NAS) was evaluated blindly by a pathologist, assessing steatosis (0–3), lobular inflammation (0–3), and ballooning (0–2). The NAS score, ranging from 0 to 8 points, is a relatively objective histological scoring system (Juluri et al., 2011).

### 2.4 Complete blood count (CBC) analysis and blood biochemical analysis (BBA)

CBC: Fresh whole blood samples should be collected in anticoagulant tubes and gently inverted 4–5 times immediately to prevent clotting prior to analysis. Then, a 20 µL aliquot of the processed sample was analyzed using the Shenzhen Mairui BC-5100 fully automated hematology analyzer and its corresponding reagent kit according to the manufacturer’s instructions.

BBA: Processed samples were analyzed according to the corresponding test requirements. Fresh whole blood samples must be collected in anticoagulant tubes and gently inverted 4–5 times immediately to prevent clotting. Fresh serum or plasma can be tested directly. Measurements of indicators such as HDL, LDL, CHOL, and TG were performed using the Mindray BS-360E fully automated biochemical analyzer and its matched reagent kits. Ensure sufficient sample volume prior to analysis (2–35 µL per indicator). If the sample volume is limited and multiple indicators need to be tested, appropriate dilution may be applied. Note that significant hemolysis may affect the results. Please refer to the instrument manual for detailed operating procedures.

### 2.5 Metabolomics analysis

Intestinal contents were collected from the cecum, snap-frozen, and extracted with methanol-chloroform. The extraction mixture was then stored in 30 min at −20 °C. After centrifugation at 20,000 g for 15 min, the supernatants were transferred into new tube to and vacuum dried. The samples were redissolved with 100 μ L 80% methanol and stored at −80 °C prior to the LC-MS analysis. In addition, pooled QC samples were also prepared by combining 10 μL of each extraction mixture.

All samples were acquired by the LC-MS system followed machine orders. Firstly, all chromatographic separations were performed using an UltiMate 3000 UPLC System (Thermo Fisher Scientific, Bremen, Germany). An ACQUITY UPLC T3 column (100 mm*2.1 mm, 1.8 µm, Waters, Milford, United States) was used for the reversed phase separation. The column oven was maintained at 40 °C. The low rate was 0.3 mL/min and the mobile phase consisted of solvent A (5 mM ammonium acetate and 5 mM acetic acid) and solvent B (Acetonitrile). Gradient elution conditions were set as follows: 0–0.8 min, 2% B; 0.8–2.8 min, 2%–70% B; 2.8–5.6 min, 70%–90% B; 5.6–6.4 min, 90%–100% B; 6.4–8.0 min, 100% B; 8.0–8.1 min, 100%–2% B; 8.1–10 min, 2%B.

A high-resolution tandem mass spectrometer Q-Exactive (Thermo Scientific) was used to detect metabolites eluted form the column. The Q-Exactive was operated in both positive and negative ion modes. Precursor spectra (70–1050 m/z) were collected at 70,000 resolution to hit an AGC target of 3e6. The maximum inject time was set to 100 ms. A top 3 configuration to acquire data was set in DDA mode. Fragment spectra were collected at 17,500 resolution to hit an AGC target of 1e5 with a maximum inject time of 80 ms. In order to evaluate the stability of the LC-MS during the whole acquisition, a quality control sample (Pool of all samples) was acquired after every 10 samples.

The samples were analyzed in a randomized order, with QC samples inserted at the beginning, middle, and end of the sequence to assess analytical reproducibility. Metabolomic profiling was performed under both positive and negative ionization modes. Peak extraction and quality control were conducted using XCMS, while metabolite identification was carried out with metaX. The identified metabolites were annotated against common functional databases, followed by quantitative analysis, sample correlation analysis, and differential analysis. The threshold for differential metabolites was set as follows: fold change (FC) ≥ 1.2 or FC ≤ 1/1.2, with a p-value <0.05 (Xia et al., 2009; Want et al., 2013). Differential metabolites were further subjected to KEGG pathway enrichment, interaction network analysis, and metabolite correlation analysis to explore their biological significance. For the ions detected by XCMS, primary identification was achieved using the open-source software metaX, which matches precursor ion m/z values to entries in public databases such as HMDB (Version 5.0) and KEGG (release 110.0, 1 April 2024), yielding preliminary identification results. However, because many isomeric metabolites share identical m/z values, primary identification alone often produces multiple candidates matches for a single ion, limiting confidence in the results. To improve accuracy, we then matched the MS/MS spectra of the sample metabolites against an in-house spectral library, obtaining higher-confidence metabolite identifications.

### 2.6 Gut microbiome analysis

The total fecal microbial DNA was obtained through the Fecal Genome DNA Extraction Kit (AU46111-96, BioTeke, China) according to the manufacturer’s instruction manual. The DNA was quantified by Qubit (Invitrogen, United States). Total DNA was amplified by PCR using the universal primer 341F/805R (341F: 5′-CCT ACGGGNGGCWGCAG-3′; 805R: 5′-GACTACHVGGG TATCTAATCC-3′). The PCR amplification conditions were pre-denaturation at 98 °C for 30 s, denaturation at 98 °C for 10 s, annealing at 54 °C for 30 s, extension at 72 °C for 45 s and 32 cycles. The final extension was at 72 °C for 10 min. The PCR product was purified using AMPure XT Beads (Beckman Coulter Genomics, Danvers, MA, United States) and quantified using Qubit (Invitrogen, United States). Qualified PCR products were evaluated using an Agilent 2,100 Bioanalyzer (Agilent, United States) and Illumina library quantitative kits (Kapa Biosciences, Woburn, MA, United States), which were further pooled together and sequenced on an Illumina NovaSeq 6,000 (PE250), provided by LC-Biotechnology Co., Ltd., Hangzhou, China.

Sequencing primers were removed from de-multiplexed raw sequences using cut adapt (v1.9). Then, Pairedend reads were merged using FLASH(v1.2.8). The low-quality reads (quality scores<20), short reads (<100 bp), and reads containing more than 5% “N” records were trimmed by using the sliding-window algorithm method in fqtrim (v 0.94). Quality filtering was performed to obtain high-quality clean tags according to fqtrim. Chimeric sequences were filtered using Vsearch software (v2.3.4) (Aebersold and Mann, 2016). DADA2 was applied for denoising and generating amplicon sequence variants (ASVs) (Callahan et al., 2016). The sequence alignment of species annotation was performed by QIIME2 (Bolyen et al., 2019) plugin feature-classifier, and the alignment database was SILVA and NT-16S. Alpha and beta diversities were calculated using QIIME2, Relative abundance was used in bacteria taxonomy. The Wilcox test was used to identify the differentially abundant genus, and significances were declared at *P* < 0.05. LDA effect size (LEfSe, LDA ≥3.0, P value <0.05) was performed using nsegata-lefse. Other diagrams were implemented using the R package (v3.4.4).

### 2.7 Statistical analysis

Data are presented as mean ± SD. Comparisons used one-way ANOVA with Tukey’s post-hoc test for multiple groups, or Student's t-test for two groups. Metabolomics and microbiota data were analyzed with multivariate statistics (PCA, PLS-DA). Correlations between ferroptosis markers, metabolites, and taxa were assessed via Spearman’s rank test. Significance was set at *p* < 0.05.

## 3 Results

### 3.1 Effects on body weight and glucose homeostasis

**FIGURE 1:**
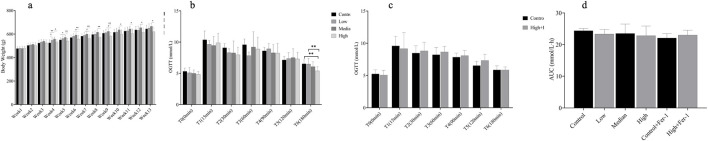
The weight of rats **(a)**, Glucose tolerance test **(b,c)** and the area under the blood glucose curve (AUC) **(d)**. * indicates *p* < 0.05; ** indicates *p* < 0.01.

Chronic 1-NP exposure did not significantly alter body weight gain compared to controls (control: 644.25 ± 30.50 g; low dose: 651.00 ± 36.15 g; Median dose: 660.00 ± 24.48 g; high dose: 620.13 ± 38.89 g at endpoint, *p* > 0.05) (Figure 1a). Rats in the control, low-dose, and medium-dose groups demonstrated a dose-dependent increase in body weight, though no statistically significant differences were observed among most of the groups. Starting from the fourth week, a statistically significant reduction in body weight was observed in high-dose rats compared to medium-dose rats. The effect of 1-NP on growth appears to follow a biphasic pattern, with low doses promoting and high doses inhibiting growth, which may be attributed to a stimulatory-like effect of low-level toxicant exposure. However, FBG levels increased progressively in exposed groups. At month 2, FBG was 5.8 ± 0.4 mmol/L in controls, 7.2 ± 0.6 mmol/L in low dose (*p* < 0.01), and 8.5 ± 0.7 mmol/L in high dose (*p* < 0.001). The oral glucose tolerance test results in the exposure groups showed that as time progressed, the dose-dependent blood glucose decline began to change after 60 min. The medium- and high-dose groups exhibited an increase rather than a decrease in blood glucose levels, reaching peak values at 120 min (Figure 1b). However, by 180 min, the blood glucose levels in the control and low-dose groups returned to levels exceeding those of the high-dose group, and this difference was statistically significant.

In the intervention groups (Figure 1c), the oral glucose tolerance test results indicated that, compared to the control + Fer-1 group, the blood glucose level in the high-dose exposure + Fer-1 group surpassed that of the control + Fer-1 group after 30 min. Although this difference was not statistically significant, it suggests that high-dose exposure may affect rat metabolism, with this effect becoming apparent after 30 min.

The area under the blood glucose curve (AUC) (Figure 1d) did not reveal significant intergroup differences, suggesting that the effects of 1-NP exposure and Fer-1 intervention on rat blood glucose are relatively complex.

The results indicated that chronic 1-NP exposure may cause certain metabolic impairments in rats, though these effects did not exhibit a clear dose-dependent relationship, suggesting that the underlying mechanisms of damage are complex. Additionally, Fer-1 intervention did not significantly ameliorate the detrimental effects induced by 1-NP.

### 3.2 Complete blood count (CBC) analysis

**FIGURE 2:**
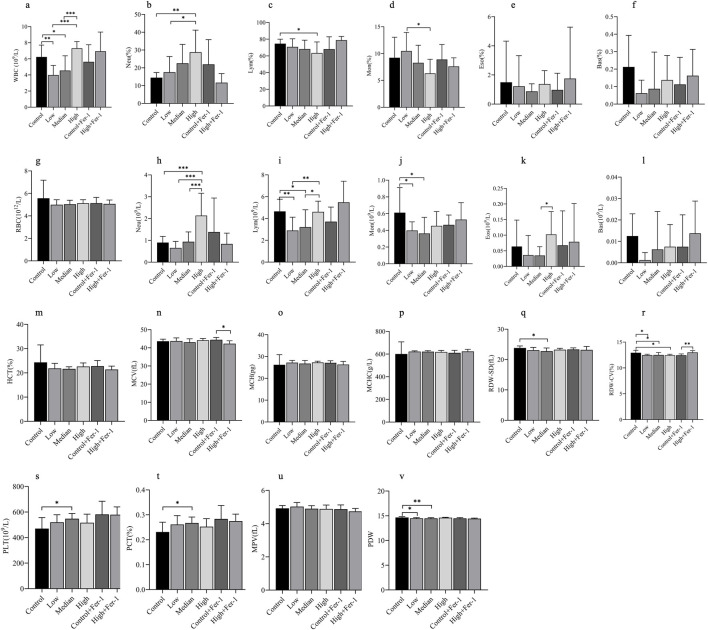
Changes in blood routine **(a–v)**. * indicates *p* < 0.05; ** indicates *p* < 0.01; *** indicates *p* < 0.001.

Prior to sacrifice, blood was collected from the canthus of the rats and subjected to complete blood count (CBC) analysis, with the results shown in the Figure 2. Statistically significant results were observed in the following indicators: WBC, Neu, Neu%, Lym, Lym%, Mon, Mon%, Eos, MCV, RDW-SD, RDW-CV, PCT, and PDW. Among these, Neu% and Lym% exhibited a dose-dependent trend, progressively increasing or decreasing from the control group to the low, medium, and high toxic exposure groups. Indicators such as WBC, Mon%, Bas%, RBC, Neu, Bas, HCT, RDW-CV, and MPV showed a gradual increase or decrease across the low, medium, and high exposure groups. The intervention effect of Fer-1 was reflected in MCV and RDW-CV. Overall, chronic 1-NP exposure can affect the immune system of rats and pose a risk of chronic inflammation. The intervention effect of Fer-1 is primarily observed in its impact on red blood cell-related indicators.

### 3.3 Changes in blood biochemistry

**FIGURE 3:**
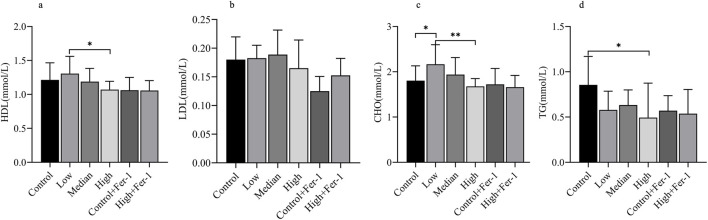
Blood Biochemistry **(a–d)**. * indicates *p* < 0.05; ** indicates *p* < 0.01; *** indicates *p* < 0.001.

Through blood biochemical analysis (Figure 3), we observed that HDL and CHO exhibited a gradual decreasing trend with increasing dosage, with a significant difference between the low-dose and high-dose exposure groups. In contrast, LDL and TG showed no consistent trends with varying exposure doses, and a significant difference in TG was only observed between the control and high-dose groups. Overall, the dose-dependent decrease in HDL across the low-, medium-, and high-dose groups, coupled with the sequential increase in LDL from the control to medium-dose groups, suggests that 1-NP exposure may adversely affect the blood lipid profile and increase the risk of abnormal lipid metabolism and cardiovascular diseases.

### 3.4 Liver pathology and NAFLD indicators

H&E staining revealed hepatocyte ballooning, lobular inflammation, and macro vesicular steatosis in 1-NP groups, absent in controls. Oil Red O confirmed lipid droplets accumulation. NAS scores were 0.63 ± 0.74 in controls, 0.50 ± 0.76 in low dose, 0.88 ± 0.99 in medium dose and 0.13 ± 0.35 in high dose, consistent with NAFLD progression. The NAS results from different dose groups exposed to toxins showed no signs of non-alcoholic fatty liver disease. However, in the Fer-1 intervention group, the NAS score in the high + Fer1 subgroup was significantly higher compared to the control + Fer-1 subgroup (*p* < 0.001), leading us to speculate that Fer-1 may exert a certain protective effect.

### 3.5 Metabolomics

**FIGURE 4:**
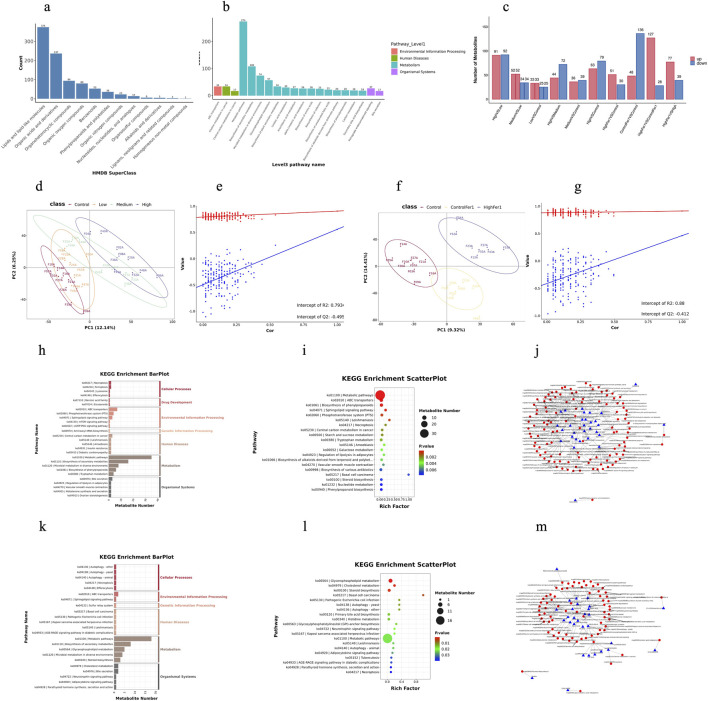
Metabolomics. **(a,b)** are identified IDMS2 metabolites were annotated with classification information from the HMDB and KEGG databases and visualized through plotting; **(c)** the number of differential metabolites identified based on secondary spectra generated by mass spectrometry detection; **(d,f)** are the PLS-DA score plots for the exposed group and the intervention group, respectively; **(e,g)** are the PLS-DA permutation test plots for the exposed group and the intervention group, respectively; **(h,k)** are hierarchical bar charts showing the primary classification and pathway names of KEGG differential metabolites for the exposed group and the intervention group, respectively; **(i,l)** are KEGG enrichment bubble charts for the toxic group and intervention group, displaying the top 20 pathways with the smallest P-values; **(j,m)** are regulatory relationship network diagrams between metabolites and pathways for the toxic group and intervention group, respectively, based on the KEGG information of differential metabolites.

This study conducted metabolomic and gut microbiota analyses on all samples. To facilitate comparison, separate analyses were performed on the control, low-, medium-, and high-dose groups exposed to 1-NP to investigate the toxic effects of 1-NP and the intervention effects of Fer-1. The detailed results of the metabolomic analysis are presented in Figure 4.

The identified IDMS2 metabolites were annotated with classification information from the HMDB (Figure 4a) and KEGG (Figure 4b) databases and visualized through plotting. According to the HMDB superclass classification rules, the metabolites were primarily clustered in Lipids and lipid-like molecules (375 compounds), Organic acids and derivatives (237 compounds), and Organoheterocyclic compounds (94 compounds). KEGG pathway analysis (Level 3) revealed that the metabolites were mainly enriched in Metabolism - Metabolic pathways (274 compounds), Metabolism -Biosynthesis of secondary metabolites (108 compounds), and Metabolism- Microbial metabolism in diverse environments (74 compounds). Other primary pathways (Level1) involved included Environmental Information Processing, Human Diseases, and Organismal Systems.

As shown in Figure 4c, the number of differential metabolites identified based on MS/MS spectra generated by mass spectrometry analysis indicates that the intervention group generally had more differential metabolites compared to the exposure dose groups, suggesting that Fer-1 intervention has a significant impact on metabolism.

PLS-DA is a supervised differential discriminant analysis method that can maximally reflect the differences between various groups. This method utilizes partial least squares regression to establish a relationship model between metabolite abundance and sample categories, enabling the modeling and prediction of samples. Additionally, a 200-iteration 7-fold cross-validation was performed on the PLS-DA results to determine whether the PLS-DA model was overfitted. In this study, the PLS-DA score plots for the exposed group and the intervention group are shown in Figures 4d,f, respectively, and the permutation test plots are presented in Figures 4e,g. The thresholds for screening differential metabolites were set as follows: FC>=1.2 or FC<=0.833, and *p*-value <0.05. As can be seen from the figure, the metabolite differences between the control group, the low-dose exposure group, and the medium- and high-dose exposure groups are significantly distinct. In the analysis of intervention samples, the differences among the control, control + Fer-1 intervention, and high-dose + Fer-1 intervention groups are evident. Meanwhile, the permutation test plot indicates that the predictive model is not overfitted.

The effects of different 1-NP exposure doses on metabolic products are reflected in the primary categories of Metabolism and Environmental Information Processing, with third-level categories including: Metabolism—Biosynthesis of Secondary Metabolites, Metabolism—Microbial Metabolism in Diverse Environments, Metabolism—Biosynthesis of Phenylpropanoids, Metabolism—Tryptophan Metabolism, Environmental Information Processing—ABC Transporters, and Environmental Information Processing—Phosphotransferase System (Figures 4h,i). The effects of the intervention group are reflected in the primary categories of Metabolism and Organismal Systems, with third-level categories including: Metabolism—Biosynthesis of Secondary Metabolites, Metabolism—Glycerophospholipid Metabolism, Metabolism—Microbial Metabolism in Diverse Environments, Metabolism—Steroid Biosynthesis, Organismal Systems—Cholesterol Metabolism, Organismal Systems—Bile Secretion, and Environmental Information Processing—ABC Transporters (Figures 4k,l). It is evident that 1-NP exposure can affect the Biosynthesis of Phenylpropanoids and Tryptophan Metabolism in rats, while Fer-1 intervention can influence Glycerophospholipid Metabolism and Steroid Biosynthesis in rats.

Based on the KEGG information of differential metabolites, regulatory network diagrams depicting the relationships between metabolites and pathways were constructed, as shown in Figures 4j,m. The key metabolite-pathway in the exposed group was Fructose 6-phosphate—Metabolic Pathways, while the key metabolite-pathway in the intervention group was Cholesterol—Metabolic Pathways.

### 3.6 Gut microbiota

**FIGURE 5:**
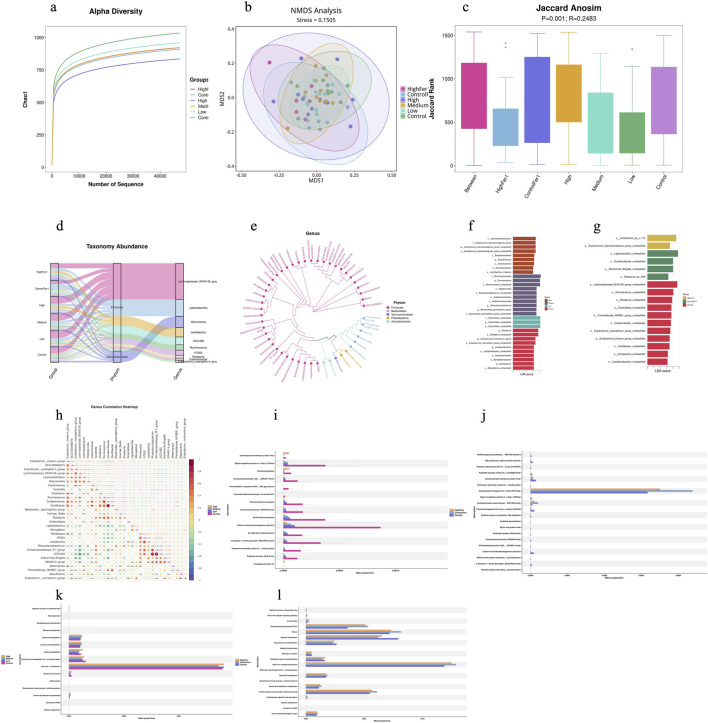
Gut Microbiota. **(a)** Alpha Diversity; **(b)** NMDS Analysis (Beta Diversity); **(c)** Jaccard Anosim (Beta Diversity); **(d)** Sankey diagram (Species composition analysis); **(e)** Genus Phylotree (Species composition analysis); **(f)** LEfSe differential analysis (Exposure Groups); **(g)** LEfSe differential analysis (Intervention Groups); **(h)** Spearman correlation heat map; **(i)** Phylogenetic Investigation of Communities by Reconstruction of Unobserved States (COG) (Exposure Groups); **(j)** Phylogenetic Investigation of Communities by Reconstruction of Unobserved States (COG) (Intervention Groups); **(k)** Phylogenetic Investigation of Communities by Reconstruction of Unobserved States (KEGG) (Exposure Groups); **(l)** Phylogenetic Investigation of Communities by Reconstruction of Unobserved States (KEGG) (Intervention Groups); Integrated analysis revealed microbiome-metabolome links: Amoebiasis with Phosphotransferase system (PTS), Starch and sucrose metabolism, and Tryptophan metabolism in exposure dose group, Histidine metabolism with Pathogenic *Escherichia coli* infection in Fer-1 intervention group. Those suggesting dysbiosis-driven metabolic shifts amplifying ferroptosis.

Sequencing yielded ∼50,000 reads/sample. Alpha diversity analysis (Figure 5a) revealed a dose-dependent negative correlation in the Chao1 index among the exposure groups, indicating reduced species richness with increasing dosage. However, no clear pattern was observed in the intervention groups. Non-metric Multidimensional Scaling (NMDS) analysis about Beta diversity (Figure 5b) showed a stress value < 0.2, indicating that the results can be effectively visualized in a two-dimensional point plot. The resulting plot demonstrates interpretability, as the distances between points representing different groups provide a meaningful reflection of the degree of variation among samples from these groups. Analysis of similarities (ANOSIM) (Figure 5c) results indicated significant differences between and within the high-dose exposure with Fer1 intervention group and the low-dose exposure group. Sankey diagram (Figure 5d) (Species composition analysis) results indicate that the two phyla with the highest abundance across different sample groups are Firmicutes and Verrucomicrobiota. The top 7 genus in relative abundance are Lachnospiraceae, Ligilactobacillus, Akkermansia, *Lactobacillus*, UCG-005, Ruminococcus, and HT002.

The Genus Phylogenetic Tree (Figure 5e) illustrates the evolutionary process of microbial communities from the phylum to genus level based on correlation analysis results. The LEfSe differential analysis (Figures 5f,g) results revealed that in the exposure groups, significant differences were observed in *Gammaproteobacteria* in the high-dose group, *Ruminococcus* in the medium-dose group, *Clostridiales* in the low-dose group, and *Eubacterium* and *Lactiplantibacillus* in the control group compared to other groups. In the intervention groups, significant differences were found in *Eubacterium xylanophilum*, *Eubacterium siraeum*, and *Lactiplantibacillus* in the control group; *Ligilactobacillus* and *Muribaculaceae* in the Control + Fer-1 group; and *Lactobacillus* and *Eubacterium coprostanoligenes* in the High + Fer-1 group compared to other groups. The correlations (Figure 5h) between different species across all samples were predominantly positive, with the strongest positive correlations observed between: Oscillibacter and Colidextribacter; UCG-005 and Phascolarctobacterium; and UCG-005 and Christensenellaceae. Based on the homologous protein annotation database developed by NCBI, comparisons with complete genome-encoded proteins revealed that Carbon monoxide dehydrogenase subunit G, Signal recognition particle GTPase, and RecB family exonuclease show relatively significant differences among various exposure dose groups (Figure 5i). In the intervention group, Formate/nitrite transporter FocA of the FNT family exhibited the most significant difference (Figure 5j). Sed on the biological metabolic pathway analysis database KEGG, the differences in microbial communities were categorized into six main groups: Metabolism, Genetic Information Processing, Environmental Information Processing, Cellular Processes, Organismal Systems, and Human Diseases. Comparative results revealed that in this study, the differences among the various exposure dose groups were attributed to processes such as Cell cycle – *Caulobacter*, Synthesis and degradation of ketone bodies, Toluene degradation, Linoleic acid metabolism, and Bisphenol degradation (Figure 5k). In the intervention groups, the differences were attributed to processes such as Other ion-coupled transporters, Bacterial chemotaxis, Protein folding and associated processing, Phosphotransferase system (PTS), and Cyanoamino acid metabolism (Figure 5l).

## 4 Discussion

This study provides comprehensive evidence that chronic 1-nitropyrene (1-NP) exposure disrupts multiple physiological systems, including glucose and lipid metabolism, immune function, liver integrity, and gut microbiota composition. Notably, these alterations were not strictly dose-linear, suggesting that 1-NP exerts complex, multifactorial biological effects that extend beyond simple toxic accumulation. By integrating biochemical, histological, hematological, metabolomic, and microbiome data, we identify several mechanistic pathways through which 1-NP may drive systemic metabolic dysfunction.

A key finding is the impairment of glucose homeostasis, demonstrated by progressive elevation of fasting blood glucose (FBG) and disrupted oral glucose tolerance. The biphasic body weight response—mild stimulation at low doses but growth inhibition at high doses—aligns with the concept of hormetic effects frequently observed with environmental pollutants (Kim et al., 2018; Agathokleous et al., 2024). Metabolomic pathway enrichment further supports metabolic disruption, particularly involving fructose 6-phosphate–centered glycolytic pathways (Furuya et al., 1982; Meurice et al., 2004), tryptophan metabolism (de Souza et al., 2025; Gabela et al., 2025), and phenylpropanoid metabolism (Dixon and Paiva, 1995). These pathways regulate energy balance, redox status, and inflammatory signaling, suggesting that 1-NP exposure perturbs core metabolic hubs rather than isolated metabolic endpoints (Gao et al., 2018; Nazar et al., 2025).

**FIGURE 6:**
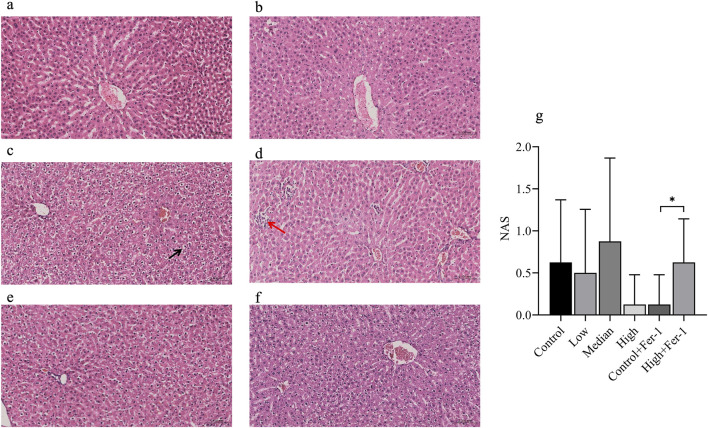
Liver pathology and non-alcoholic fatty liver disease index. **(a)** Control; **(b)** Low 1-NP Group; **(c)** Media 1-NP Group; **(d)** High 1-NP Group; **(e)** Control + Fer-1 Group; **(f)** High + Fer-1 Group; **(g)** NAS index at 6 Groups *The red arrow indicates a small amount of inflammatory cell infiltration, while the black arrow points to hepatocellular ballooning degeneration.

Lipid metabolism was also significantly disturbed. The dose-dependent reductions in HDL and total cholesterol, combined with altered LDL patterns (Li F. et al., 2020), indicate dyslipidemia and elevated cardiovascular risk (Chen et al., 2024; Qigang et al., 2024). Although the levels of cholesterol and triglycerides in the serum decreased, which was inconsistent with the expected case of hyperlipidemia, similar phenomena were also observed in some studies due to the varying toxicities of different toxins (Puri et al., 2007; Begriche et al., 2011). Liver histopathology confirmed hepatocyte ballooning, lobular inflammation, and microvascular steatosis, consistent with NAFLD-like progression (Choi Y.-H. et al., 2023; Wu et al., 2025). Interestingly, NAS scores exhibited a non-monotonic trend, with a slight decrease at the high-dose level (Figure 6g). This non-linear response suggests a toxicological shift: while low doses promote steatosis, high-dose 1-NP may trigger severe cellular stress—such as oxidative damage or ferroptosis—that hinders lipid droplet accumulation, thereby lowering the NAS score despite qualitative increases in hepatotoxicity (Kleiner et al., 2005; Vandenberg et al., 2012). Although these scores remained below overt pathological thresholds, the accompanying metabolomic evidence revealed significant disorders in glycerophospholipid and steroid metabolism (Nicholson et al., 2002). These findings highlight a “sub-clinical” metabolic reprogramming that precedes histological damage, emphasizing the importance of integrating molecular profiling with traditional pathology to evaluate the threshold effects of 1-NP (Zhu et al., 2025).

Another critical observation is the immune perturbation associated with PAHs (Yu Y. Y. et al., 2022), particularly following chronic 1-NP exposure. Hematological alterations in WBC, neutrophils, lymphocytes, and monocytes, some following dose-dependent trends, suggest a shift toward a chronic inflammatory state. Increased neutrophil percentage and reduced lymphocyte percentage are characteristic hallmarks of pollutant-induced inflammatory stress. These immune changes parallel the hepatic inflammatory lesions observed histologically, indicating that metabolic and immune toxicities are tightly interlinked (Burchiel and Luster, 2001).

Gut microbiota analysis revealed pronounced dysbiosis, including reduced alpha diversity and significant shifts in key taxa such as Gammaproteobacteria, Ruminococcus, and Clostridiales. These microbial alterations corresponded with changes in microbial functional genes (e.g., carbon monoxide dehydrogenase subunit G, formate/nitrite transporter FocA) and KEGG pathways related to xenobiotic degradation, lipid metabolism, and bacterial chemotaxis. The microbiome–metabolome integration further highlighted the involvement of phosphotransferase (PTS) systems, starch and sucrose metabolism, and tryptophan metabolism, suggesting that dysbiosis directly contributes to host metabolic impairment. The coupling of microbial functional shifts with host metabolic pathways provides mechanistic insight into how 1-NP disrupts systemic metabolic networks through both direct toxic effects and microbiota-mediated mechanisms.

Although Fer-1, a ferroptosis inhibitor, altered certain metabolic and microbiome parameters, its overall protective effect was limited (Deng et al., 2021). Fer-1 primarily modulated pathways related to glycerophospholipid metabolism (Chang et al., 2025), steroid biosynthesis (Jiang et al., 2024; He et al., 2025), cholesterol metabolism (Lee et al., 2021; Yang et al., 2025), and several bacterial functional categories but failed to reverse major physiological endpoints such as impaired glucose tolerance or liver pathology. This suggests that the toxic effects of 1-NP are not solely driven by ferroptosis but likely involve multi-pathway interactions, including oxidative stress, inflammatory activation, metabolic pathway disruption, and microbiome dysbiosis. The limited efficacy of Fer-1 therefore reinforces the complexity of the underlying mechanisms and underscores the need for interventions targeting multiple biological pathways.

Collectively, these findings position 1-NP as a potent metabolic disruptor capable of simultaneously altering host metabolism, immunity, hepatic function, and gut microbiota structure (Wang M. et al., 2023). The identification of key metabolic nodes—particularly fructose 6-phosphate, tryptophan metabolism, and glycerophospholipid pathways—provides potential mechanistic anchors for future studies. Moreover, the microbiome–metabolome interactions observed here highlight the importance of considering host–microbial crosstalk in environmental toxicology research. Understanding these interconnected pathways is crucial for elucidating how ubiquitous nitro-PAHs contribute to metabolic diseases such as NAFLD, diabetes, and cardiovascular disorders.

Several limitations of this study should be acknowledged. First, although multiple doses of 1-NP were examined, the exposure duration and environmental relevance of the selected concentrations may not fully represent real-world human exposure scenarios. Second, while metabolomics and microbiome analyses provided broad insights into pathway perturbations, the causal relationships between microbial shifts, metabolic alterations, and organ-specific toxicity remain inferential and require targeted validation. Third, although sex differences in 1-NP toxicity have been reported in various systems (Li et al., 2019; Li J. et al., 2020), no consistent pattern has emerged, in contrast to the significant sex-dependent differences commonly seen in the hepatotoxicity of other organic toxicants (Hari et al., 2024). Despite this, the present study focused solely on male rats, without evaluating potential sex differences in susceptibility. In addition, Fer-1 intervention was administered using a single dosage regimen, which may have limited the ability to detect protective effects or dose–response interactions. Finally, although the integrated multi-omics approach revealed key pathways of interest, further mechanistic experiments—such as gene expression analyses, enzyme activity measurements, or germ-free/antibiotic-treated animal models—are needed to establish direct molecular mechanisms underlying 1-NP–induced metabolic dysfunction.

This study demonstrates that chronic exposure to 1-nitropyrene induces multifaceted metabolic toxicity characterized by impaired glucose regulation, dyslipidemia, immune disturbances, liver injury, and gut microbiota dysbiosis. These effects were supported by converging evidence from physiological measurements, serum biochemistry, histopathology, metabolomics, and microbial community analyses. Key metabolic pathways—including fructose 6-phosphate–centered glycolysis, tryptophan metabolism, glycerophospholipid metabolism, and ABC transporter activity—were significantly altered, reflecting widespread disruption of host metabolic networks. Integrated microbiome–metabolome analysis further revealed strong associations between dysbiosis and metabolic pathway perturbations, suggesting that microbial shifts contribute to the systemic toxicity of 1-NP. Although Fer-1 intervention modulated certain metabolic and microbial signatures, it did not substantially mitigate the major toxic outcomes, indicating that 1-NP–induced damage involves mechanisms beyond ferroptosis alone. Overall, our findings highlight 1-NP as a potent environmental metabolic disruptor and underscore the need for deeper mechanistic studies to understand its contribution to metabolic diseases and to develop effective preventive strategies.

## Data Availability

The original contributions presented in the study are included in the article/supplementary material, further inquiries can be directed to the corresponding author.
